# Antimicrobial Peptide Mimics for Clinical Use: Does Size Matter?

**DOI:** 10.3389/fimmu.2022.915368

**Published:** 2022-05-26

**Authors:** Johan Svenson, Natalia Molchanova, Christina I. Schroeder

**Affiliations:** ^1^ Cawthron Institute, Nelson, New Zealand; ^2^ The Molecular Foundry, Lawrence Berkeley National Laboratory, Berkeley, CA, United States; ^3^ Center for Cancer Research, National Cancer Institute, National Institutes of Health, Frederick, MD, United States

**Keywords:** antimicrobial peptides, antibiotic, synthetic mimic, amphiphilic, clinical development, peptidomimetics, resistant bacteria

## Abstract

The search for efficient antimicrobial therapies that can alleviate suffering caused by infections from resistant bacteria is more urgent than ever before. Infections caused by multi-resistant pathogens represent a significant and increasing burden to healthcare and society and researcher are investigating new classes of bioactive compounds to slow down this development. Antimicrobial peptides from the innate immune system represent one promising class that offers a potential solution to the antibiotic resistance problem due to their mode of action on the microbial membranes. However, challenges associated with pharmacokinetics, bioavailability and off-target toxicity are slowing down the advancement and use of innate defensive peptides. Improving the therapeutic properties of these peptides is a strategy for reducing the clinical limitations and synthetic mimics of antimicrobial peptides are emerging as a promising class of molecules for a variety of antimicrobial applications. These compounds can be made significantly shorter while maintaining, or even improving antimicrobial properties, and several downsized synthetic mimics are now in clinical development for a range of infectious diseases. A variety of strategies can be employed to prepare these small compounds and this review describes the different compounds developed to date by adhering to a minimum pharmacophore based on an amphiphilic balance between cationic charge and hydrophobicity. These compounds can be made as small as dipeptides, circumventing the need for large compounds with elaborate three-dimensional structures to generate simplified and potent antimicrobial mimics for a range of medical applications. This review highlight key and recent development in the field of small antimicrobial peptide mimics as a promising class of antimicrobials, illustrating just how small you can go.

## Introduction

**Figure 1:**
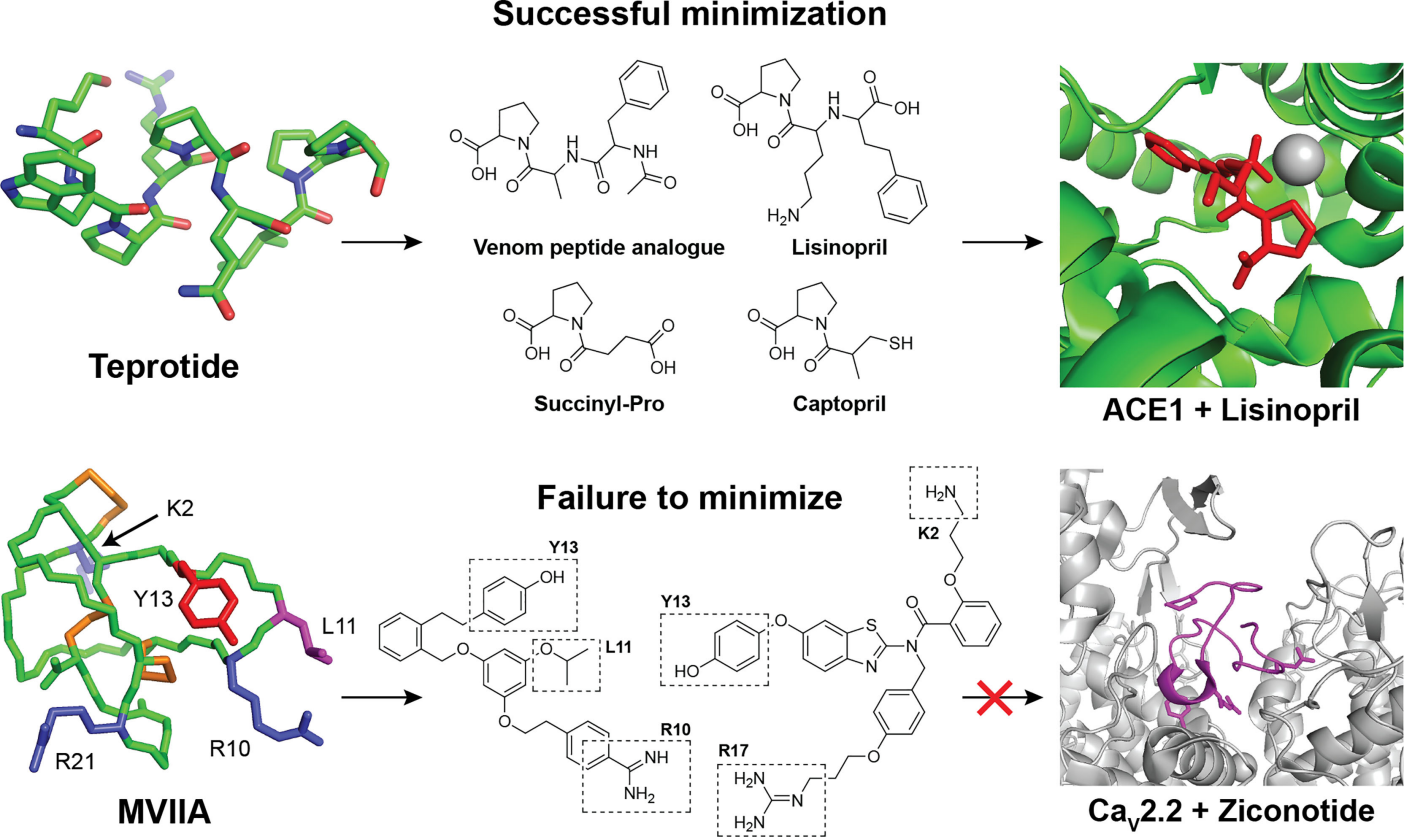
Examples of successful and unsuccessful attempts to minimize natural peptide leads during drug development. **Top row**: The natural teprotide peptide from the snake *Bothrops jararaca* is a potent inhibitor of the angiotensin-1-converting enzyme (ACE-1) (7). The native nonapeptide can be effectively truncated and modified to yield a range of simplified and optimized analogs with optimal binding to ACE-1[PDB: 1J36 (8)] (9). **Bottom row**: The highly complex and bioactive MVIIA peptide [PDB: 1TTK (10)] from the marine snail *Conus magus* have therapeutic value in the treatment of neuropathic pain by inhibiting N-type (Ca_v_2.2) voltage-gated calcium channels [PDB: 7VFW (11)] (12). Despite extensive analog development and minimization efforts, no improved leads have been reported and the final marketed drug Ziconotide is identical to the native venom peptide (13).

Therapeutic peptides have been used by humans for more than a century, since the first use of insulin in the 1920s and there are now nearly 100 approved peptide drugs on the market used for the treatment of a range of medical conditions (1). In humans, peptides are often produced and used endogenously to respond to altered physiological conditions and we have effective means to regulate their plasma concentration and half-life *via* different biochemical pathways (2, 3). While these biological systems allow the body to manage homeostasis efficiently, the natural susceptibility of innate immune peptides to enzymatic degradation represents a hurdle which combined with poor oral bioavailability hampers the introduction and developments of new peptide drugs (2, 4). To combat these challenges, modified peptides may be generated incorporating unnatural elements and truncated sequences to tune the ADME-properties (5, 6). However, for some biological targets, successful treatment depends on native peptides with elaborate structures. These complex native structures are essential for optimized target receptor binding and leads to increased production costs (**Figure 1**) (1, 14, 15).

Peptide-based drugs are used to target both intra- and extracellular targets, ion channels, GPCRs, and a variety of enzymes. Currently, the majority of new peptides entering the market are developed as treatments for the major therapeutic challenges including cardiovascular disorders, cancer, and metabolic diseases (1, 2, 16). Microbially produced peptides such as gramicidin, colistin, daptomycin and the lipoglycopeptide vancomycin are further examples of peptides that can be used to combat infectious diseases (17). Despite the success of these microbially derived peptide antibiotics, the advancement of the many endogenously produced defensive antimicrobial peptides (AMPs) in higher organisms have yet to deliver an impact on the drug market (17, 18).

AMPs represent a key component of the innate defence system involved in the initial rapid response to pathogenic intruders (3, 19, 20). AMPs are generally amphiphilic and many rapidly target microbes at the membrane level causing membrane disruption *via* modes of action less likely to cause resistance (18, 19, 21, 22). Faced with the plethora of naturally producing organisms, the composition and structure of native AMPs differs across species but falls within three groups based on their secondary structure. AMPs are generally short, and commonly adopts either an α-helical or a β-sheet structure with additional examples of extended and random-coil structured AMPs as well (23, 24). Driven by an urgent need to develop effective antimicrobials with novel modes of action due to the emergence of antimicrobial resistance (25), AMPs have been extensively studied and probed for their potential as the next generation antibiotics (18). Well-studied examples include the defensins, LL-37, lactoferricin, indolicidin and magainin (18, 26, 27). With numerous AMPs in clinical trials, it is expected that we will see this class of direct killing and/or immunomodulating compounds available for clinical infection management in the future (18, 24, 26).

The majority of the innate AMPs display highly defined secondary structures and the peptidic nature of these endogenous compounds is hampering the therapeutic development while the dependence of native sequences also results in high production costs (17). AMPs are further faced with obstacles associated with challenging pharmacokinetics, off-target toxicity, low metabolic stability, short half-life, and poor oral bioavailability (4, 28). As such, synthetic innate AMPs and their derivatives have not yet lived up to their heralded potential as the next generation antibiotics (18, 28–30).

Regardless of origin and structural class, the vast majority of AMPs carries a net positive charge (+2 to +11), balanced by a significant number of hydrophobic residues enabling an amphiphilic bioactive structure (31). Recent studies have illustrated that isolating these two functional components allows for the generation of much shorter AMPs and synthetic mimics thereof not adhering to the well-defined secondary structures found in nature (6). The observation that the key requirement for antibacterial activity is sufficient cationic charge balanced by hydrophobic elements has recently allowed the design of diverse AMP mimics as small as dipeptides with maintained and even improved bioactivity over their native counterparts (32–34). These smaller, and more “drug-like” AMP mimics come with several advantages, such as proteolytic resistance (35), potential for oral bioavailability (36, 37) and significantly lower production costs compared to the larger native AMPs (27, 28, 38, 39). The current report describes the state of the art of transferring the AMP pharmacophore onto alternative molecular scaffolds to yield simpler, yet more efficient antimicrobial leads (40). In addition, it answers the questions “How small can you go?” and “Does size matter?” in the search for leads that may offer alternatives to the native AMPs for combating resistant infections in the future.

## Making AMPs Shorter

One of the main developmental barriers of the numerous natural AMPs towards their clinical use is the need for long native sequences and a reliance on a maintained helicity for a high bioactivity (28, 41). This dependence is costly and it presents synthetic challenges on large scale as these native sequences also require folding into their native bioactive secondary structure after initial synthesis (42). Furthermore, their size represent an obstacle for oral administration, targeted delivery (28) and they are prone to proteolytic degradation. This is due to their high content of charged and hydrophobic residues, which dictates the process of binding to the active site of many proteolytic enzymes (35, 43).

**Figure 2:**
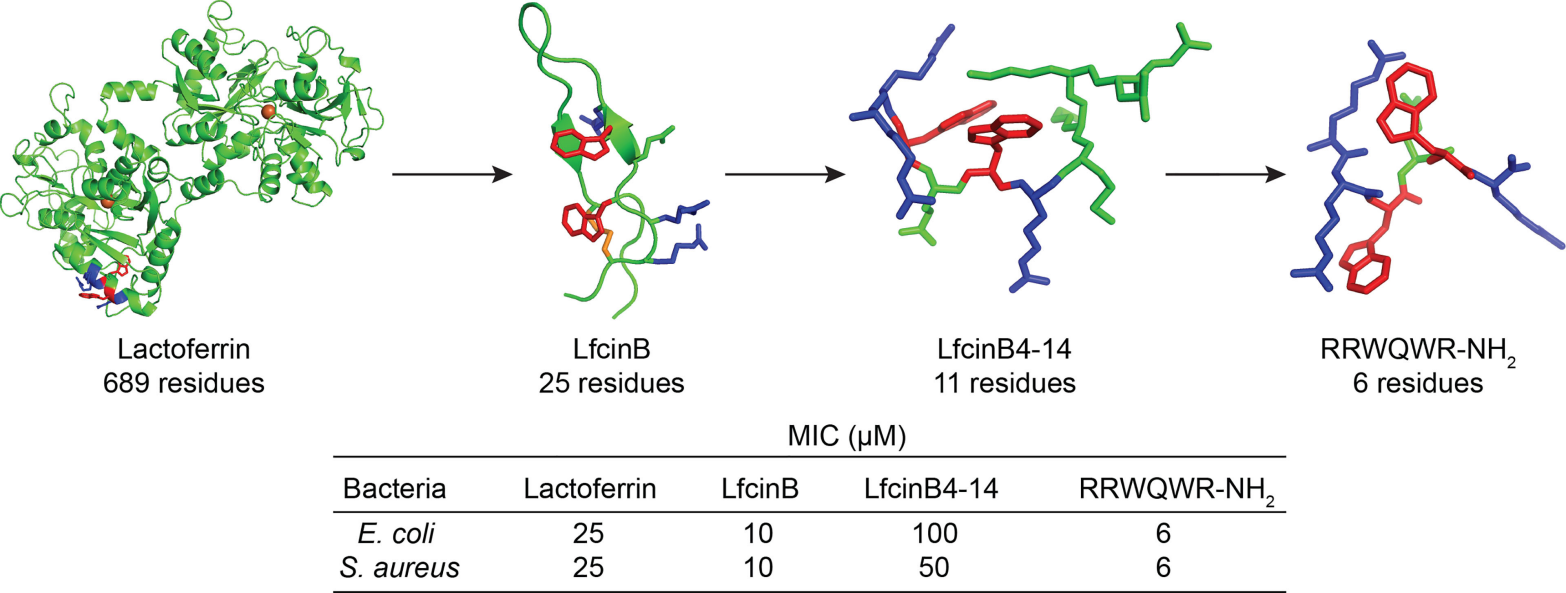
Successful truncation of the native lactoferrin protein, 689 residues, [PDB: 1BLF (44)] *via* LfcinB, 25 residues [PDB: 1LFC (45)], LfcinB4-14, 11 residues [PDB: 1Y5C (46)], to the antibacterial RRWQWR-NH_2_ motif (created and minimized in Pymol). Arg – red, Trp – blue, iron – orange ball, and disulfide bond – orange stick). The N-terminal cyclic lactoferricin peptide is formed upon digestion and linearization and additional truncation of lactoferricin yields shorter sequences with maintained antimicrobial activity. MIC-values taken from Svenson *et al. (* 47) and Tomita *et al. (* 48).

The most obvious approach to overcome the challenges associated with size is to attempt to make the peptides smaller and this can be achieved by identifying key structural features and trimming away excessive structures (1). In the field of AMPs however this has only been successfully attempted for a limited number of peptides and clinical leads. For notable AMP examples, such as LL-37, human β-defensins, indolicidin (omiganan) and magainin (pexiganan) only minor variation to the native sequence, if any, have been incorporated (24). In contrast, research on the iron binding lactoferrin protein and its antimicrobial cyclic degradation product lactoferricin, have highlighted that most of the antimicrobial activity can be maintained in linear N-terminal fragments (**Figure 2**) (49). This discovery has led to several shorter linear lactoferrin-derived lead peptides in clinical development such as hlf1-11, PXL01 and PXL150 (24, 26, 50, 51).

**Figure 3:**
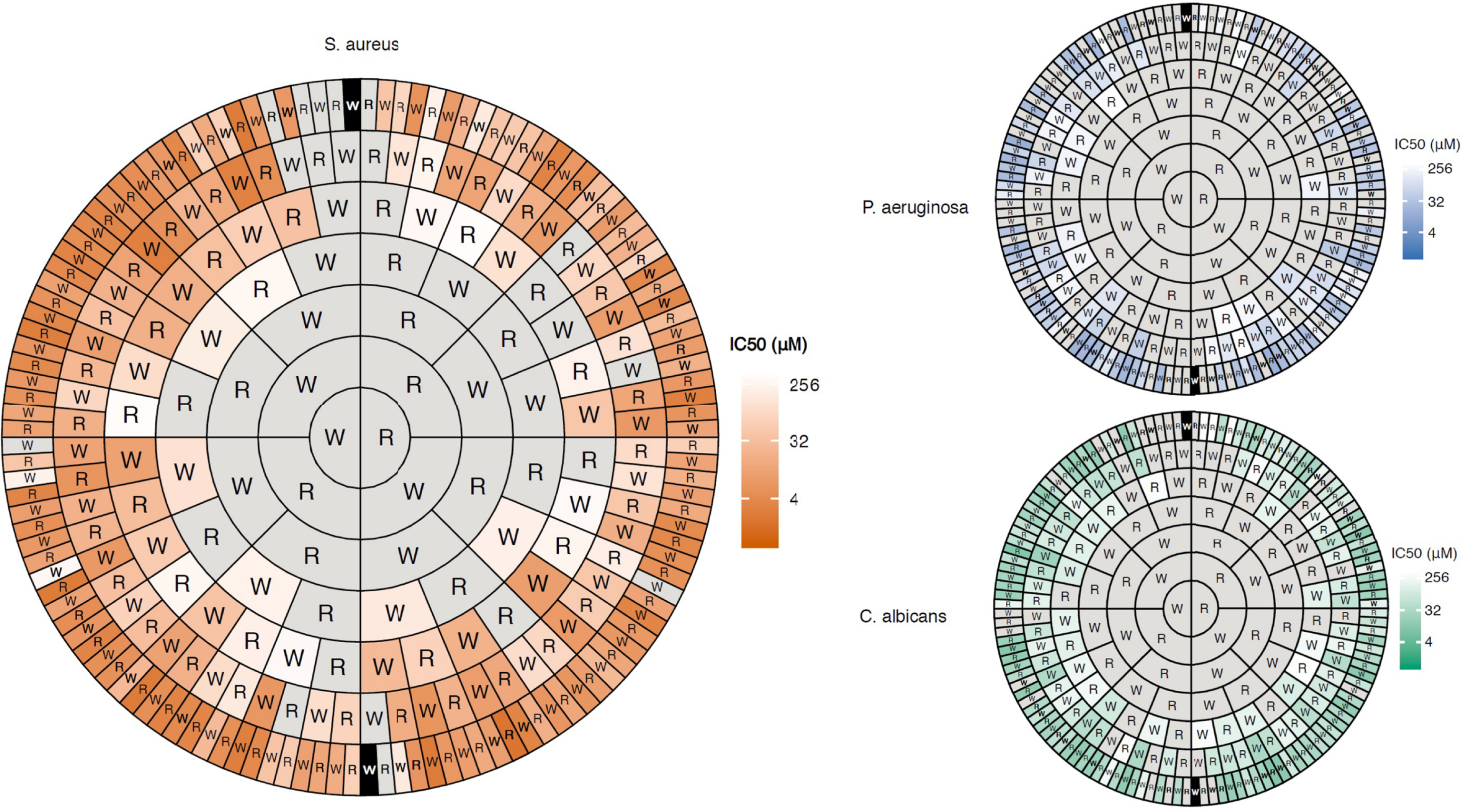
Concentric ring chart representation (Harris-Clark diagram) of the antimicrobial activity of all possible peptides comprised of W and R up to 7 residues long recently developed by Dobson and co-workers (56). The diagram highlights how the antimicrobial activity of the peptides increases with peptide length and that how most peptides are active as heptapeptides if sufficient R and W residues are combined in a non-sequence dependent manner. The data also shows how the compounds display a higher activity (shorter peptides required) against Gram-positive bacteria compared to Gram-negative ones and fungi due to differences in cell wall and membrane composition. Figure reproduced from Clark *et al. *(56) Commun. Biol. 2021 Vol. 4 Issue 1 Pages 1-14 with permission.

The helical N-terminal 15 residue fragment of bovine lactoferricin FKCRRWQWRMKKLGA was studied in detail by Svendsen and co-workers (52, 53). In a series of truncation and alanine scan experiments they showed that this sequence could be significantly improved by amino acid replacement and incorporation of unnatural hydrophobic amino acids as replacement of crucial tryptophan residues (33). By combining these findings with the early identification of an antibacterial RRWQWR motif inside lactoferricin by Tomita and co-workers (48), they embarked upon attempting to further minimize the hexapeptide to generate more “drug-like” compounds (54). By focussing on only using arginine and tryptophan and synthetic amino acids they showed across a series of key papers that a high antimicrobial activity could be obtained once a “sufficient” number of cationic residues and hydrophobic elements were assembled (32, 54, 55). The order of assembly appeared to only play a minor role (32) and this was recently verified in an elegant and extensive study by Dodson and co-workers who assembled and evaluated all (252 out of 254) possible RW peptides up to seven amino acids in length (56). As visually presented in **Figure 3**, weak activity against Gram-positive bacteria is seen for some tetrapeptides while almost all hexa- and heptapeptides display a high activity, seemingly independent of sequence, provided sufficient charge and hydrophobicity is provided (**Figure 3**).

## The 2 + 2 Pharmacophore

**Figure 4:**
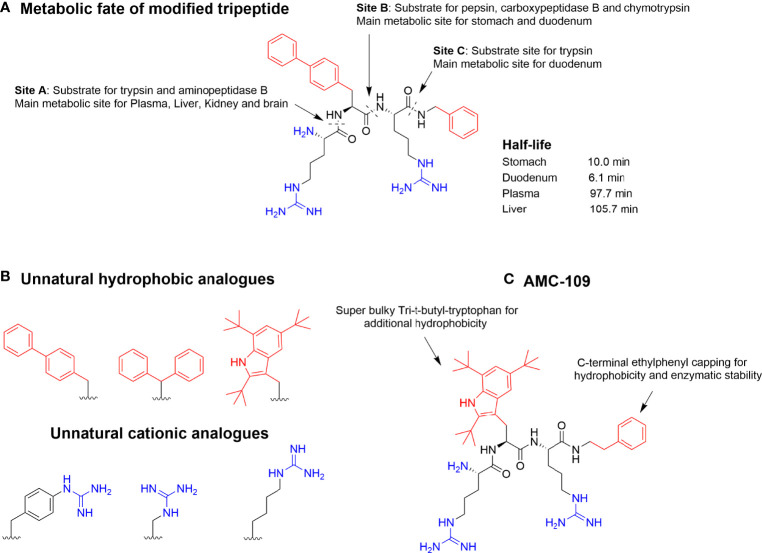
**(A)** Metabolic susceptibility of a short, modified, and highly active antimicrobial tripeptide in selected key metabolic compartments despite incorporation of bulky unnatural amino acids (47). **(B)** Incorporation of optimized unnatural cationic and hydrophobic amino acids can be used to steer the metabolic stability together with different C-capping strategies. **(C)** Optimized lead AMC-109 (previously LTX-109) currently developed by Amicoat AS (Norway) for the eradication of microbial biofilms (62).

To further try to identify the minimal antimicrobial motif of these short AMPs, C-terminal modifications were performed which allowed an additional hydrophobic element to be incorporated (32). This was highly successful and by using C-terminal benzyl esters, peptides such as WRW-OBzl and RW-OBzl (Minimal inhibitory concentrations (MIC) against *S. aureus* 5 and 25 μg/mL, respectively) were generated with the C-terminal group acting as a second hydrophobic residue (32). Through these studies it became clear that the minimum antibacterial motif was at least two positive charges balanced by two units of hydrophobicity (32). Subsequently incorporated bulky unnatural amino acids could be used to increase the bioactivity further towards low micromolar bioactivity against a range of pathogenic bacteria and fungi (57). Despite the tripeptidic nature of these compounds, they strongly associate with plasma proteins (58–60) and ex-vivo metabolism studies using organ extracts highlighted significant enzymatic susceptibility (47). This rapid degradation was ascribed high affinity interactions with the fringing S1 and S1’ binding pockets due to the combination of cationic and hydrophobic residues and illustrated how the natural functionalities needed for bioactivity also generated good substrates for serine proteases and pepsin (35, 61). Reducing the snug fit to enzymatic binding pockets by incorporating “superbulky” hydrophobic residues or cationic analogs that are either longer or shorter than arginine effectively evades enzymatic degradation (36). Following this development, several highly active and stable tripeptides have been generated with LTX-109 (now AMC-109) being the most advanced in clinical development (**Figure 4**) (6, 63, 64). AMC-109 represent a fast-acting, broad-spectrum, antimicrobial AMP drug candidate and is currently undergoing development by Amicoat (62).

Attempts to further refine the 2 + 2 pharmacophore have shown that it is more complex than initially thought and both the basicity of the cationic residues (34) as well as the shape and volume of the hydrophobic moieties are critical for the bioactivity of the final peptides (65). Several studies illustrated that guanidine groups can promote increased hydrogen bonding interactions with the phospholipids of the target microbial membrane, in comparison with amines and quaternary ammonium groups (6, 66). Dissection of the hydrophobic contribution into a minimal hydrophobic volume (65) and its placement (39) instead of a number of hydrophobic residues also generates a more accurate understanding of the essential structural requirements for high antimicrobial activity (65). While the initial “2+2” pharmacophore remains quite accurate for linear peptides, parallel and independent developments have made it apparent that other potent small AMPs and mimics thereof can be prepared using alternative scaffolds to generate potent leads (40). These tailormade synthetic mimics may offer advantages over natural sequences and they have been praised as important classes of compounds for eliminating both multidrug-resistant and pandrug-resistant bacterial isolates (18). The following section will summarize the development of the classes of small (<1000 Da) amphiphilic AMP mimics (**Table 1**) inspired by the natural cationic AMP pharmacophore which has yielded several leads in late stage clinical development (40). The focus lies on small antimicrobial mimics and our review will not cover immunomodulation (40), larger oligomeric assemblies or polymers which have been reviewed elegantly elsewhere (28, 30, 80–82).

**Table 1:** Major scaffold types used to generate small bioactive AMP mimics adhering to the 2 + 2 pharmacophore.

Scaffold	General structure^1^	Key references
**α-peptides**	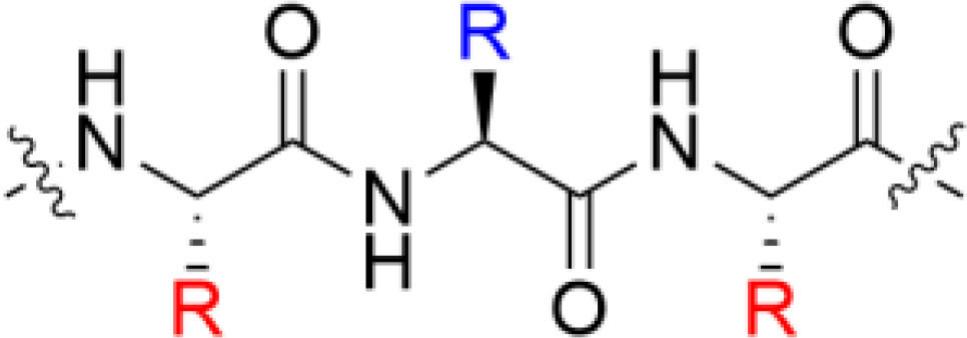	(6, 32, 56, 57)
***β*-Peptidomimetics**	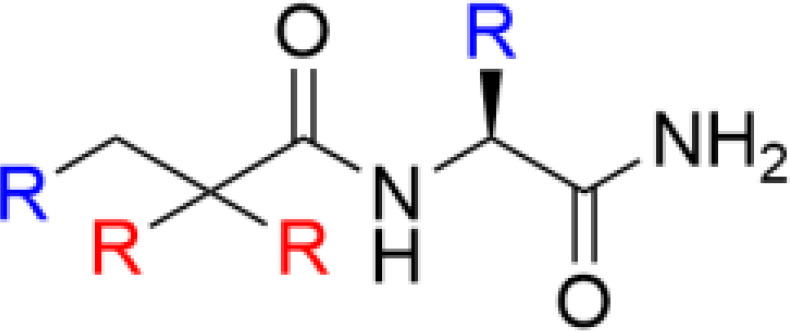	(67, 68)
**2,5-diketopiperazines**	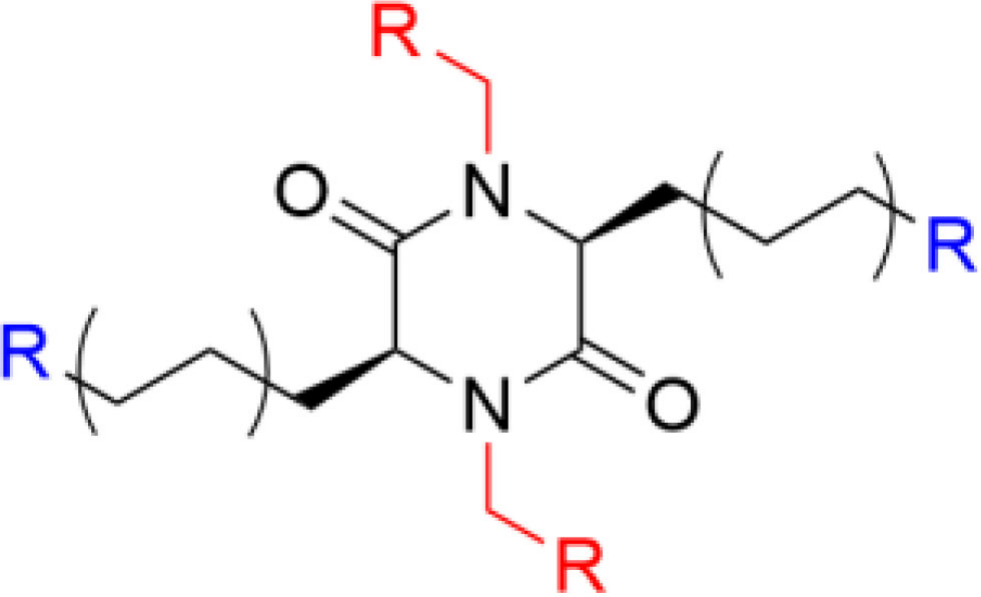	(65, 69)
**Lipopeptides**	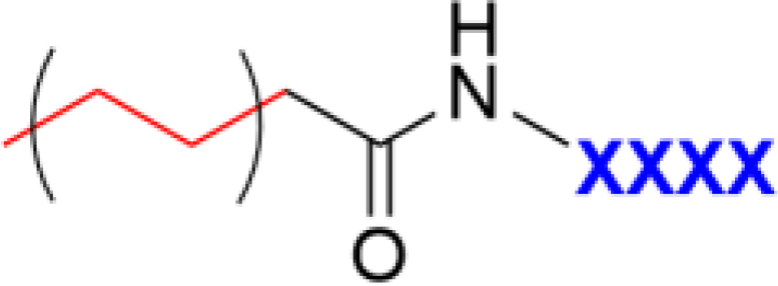	(70–72)
**Peptoids**	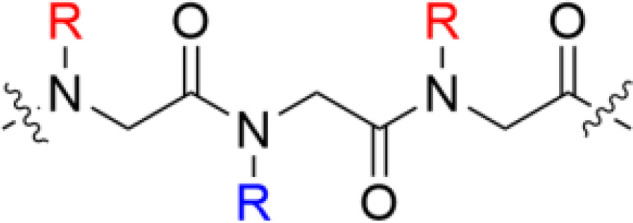	(73)
**Bile acid**	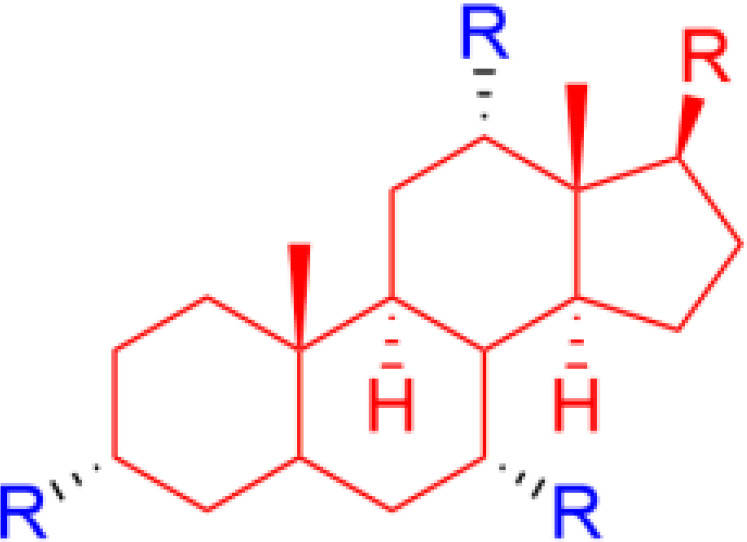	(74–76)
**SMAMPs**	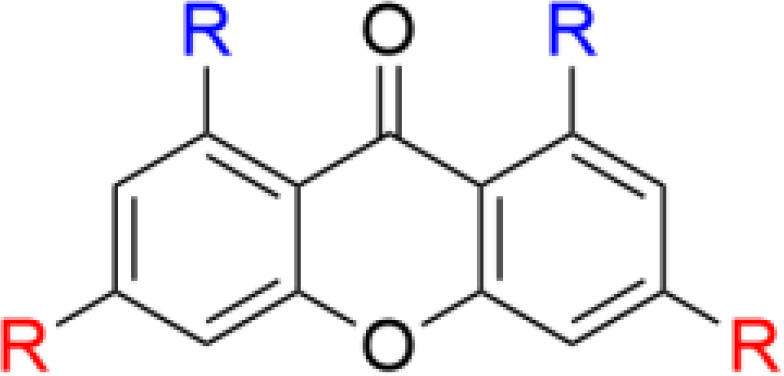	(39, 40, 77–79)

^1^Blue denotes cationic group (R) or residues (X) and red denotes bulky hydrophobic residue. The SMAMP structure is an example rather than a general scaffold as numerous different hydrophobic cores have been used for these compounds.

## *β*-Peptidomimetics and *β^2,2^*-Amino Acid Derivatives

**Figure 5:**
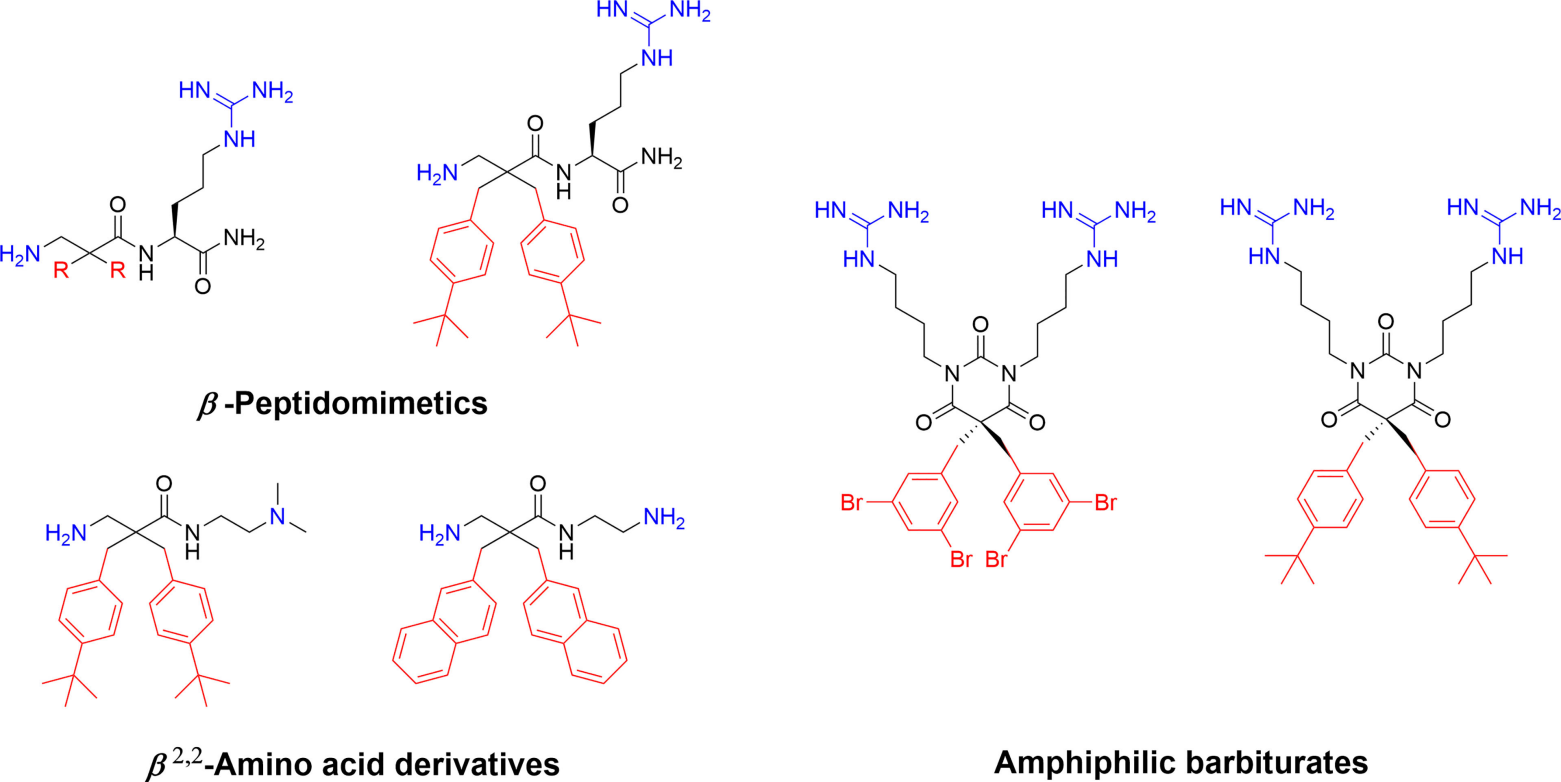
Examples of highly active antimicrobial *β*-peptidomimetics, *β* ^2,2^-amino acid derivatives and amphiphilic tetrasubstituted barbiturates develop by Strøm and co-workers (67, 68, 85). The peptidomimetics illustrate how the AMP pharmacophore effectively can be transferred to smaller scaffolds.

Recent developmental work by the Strøm lab, initially involved in the key fundamental 2 + 2 pharmacophore discovery, focussed on generating simpler AMPs mimics. By transferring the crucial substituents onto an achiral lipophilic 3-amino-2,2-disubstituted propionic acid scaffold they produced a library of *β*-peptidomimetics *(* 67). Two identical hydrophobic residues were introduced and a substantial antibacterial effect was observed for the bulkier residues, in particular for substituents incorporating one or two t-butyl substituents in analogy to the AMC-109 drug lead (63). A strong link between overall hydrophobicity and antibacterial effect was observed and the compounds were shown to display both low haemolytic activity and resilience towards chymotryptic degradation over 48 h (67). The cationic requirement was initially provided by an arginine residue at the N-terminus but studies on only the *β* ^2,2^-amino acid scaffold established that two essential cationic group could be included as N and C-terminal substituents, generating smaller compounds (68). These *β* ^2,2^-amino acid derivatives could be made with significant design freedom with active compounds fulfilling both the 2 + 2 pharmacophore and the Lipinski rules for orally bioavailable drugs (68, 83). The theoretical oral uptake was calculated and also studied experimentally for selected compounds using a phospholipid vesicle-based barrier designed to mimic the intestinal epithelia (68, 84). The permeability studies suggest a moderate oral absorption in humans, which is in alignment with similar experimental studies on modified tripeptides and also highlights how the theoretical uptake models are not necessarily the optimal method for assessing permeation for this class of compounds (37). The effect of a range of both hydrophobic and cationic elements and halogenation have been evaluated in this scaffold and the reported structural observations are similar to those observed for the linear tripeptides (6) with guanidine groups performing better as cationic groups and an overall pattern of dependency on “sufficient hydrophobic bulk” for optimal activity (**Figure 5**) (86). The compounds have been extensively evaluated as antimicrobials and demonstrated to be highly active against many multi-resistant clinical isolates and also intracellular pathogens such as *Chlamydia pneumoniae* (86, 87).

The natural marine antimicrobial eusynstyelamides have been isolated from both the marine Arctic bryozoan *Tegella cf. spitzbergensis (* 88) and the Australian ascidian *Eusynstyela latericius (* 89). The eusynstyelamides are composed of a central five-membered dihydroxybutyrolactam ring naturally substituted with two 6-bromo-indoles and two cationic sidechains, containing both guanidine and amine groups (88). As such they are naturally adhering to the AMP pharmacophore and a recent study by Strøm and co-workers describes how the dihydroxybutyrolactam core can be replaced with a simpler achiral barbiturate ring which allows for rapid generation of synthetic analogs (**Figure 5**) (85). These tetrasubstituted barbiturates were shown to be highly antimicrobial with good cellular selectivity over human erythrocytes and active towards resistant clinical isolates. Introduction of “superbulky” hydrophobic sidechains generated more active analogs compared to the natural compounds. A lead compound was evaluated *in vivo* in a murine peritonitis model and a single intraperitoneal injection (1.4 mg/kg) resulted in a 98% reduction of the bacterial load of *Klebsiella* pneumoniae (85).

## 2,5-Diketopiperazines

**Figure 6:**
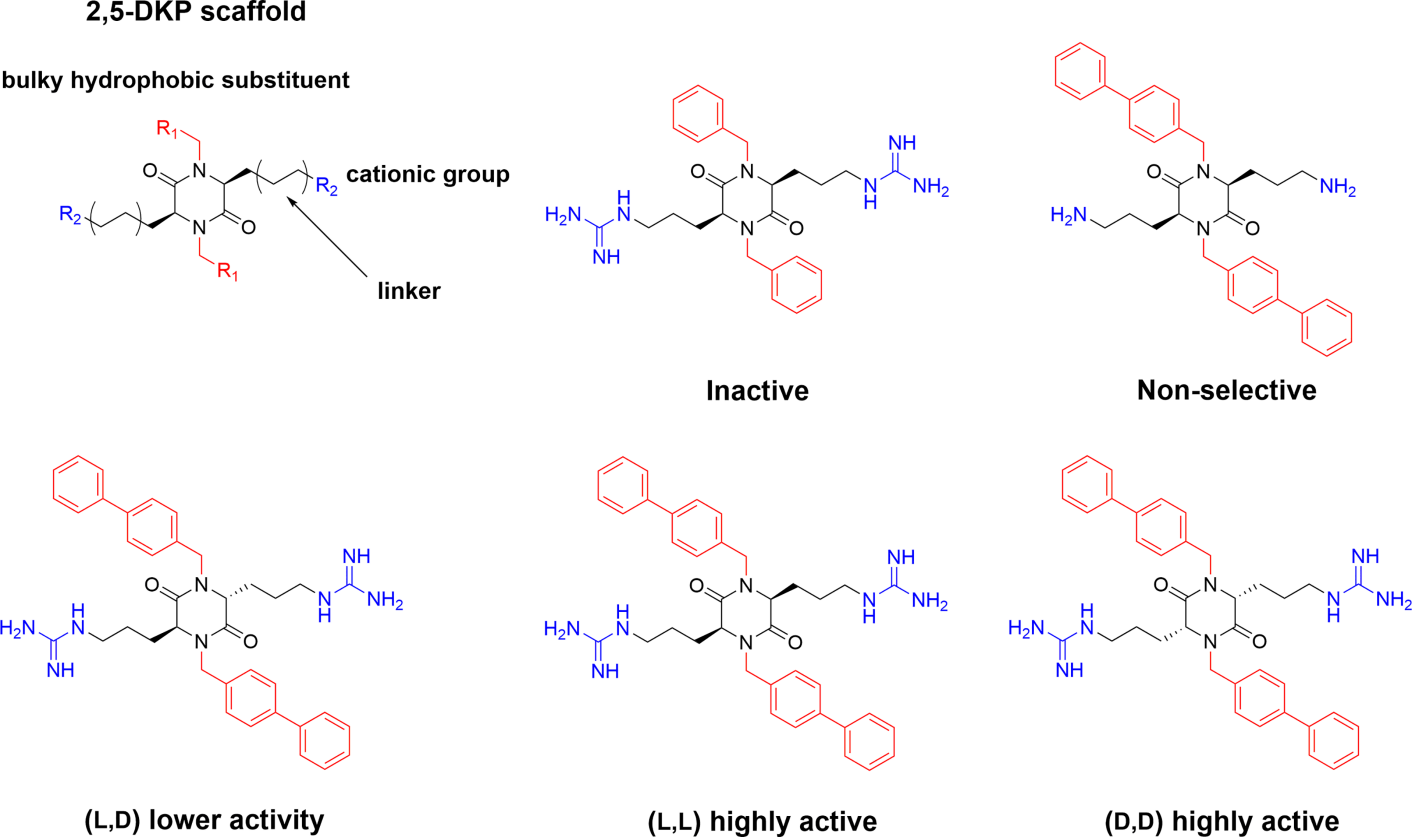
Design of the antimicrobial 2,5-DKP scaffold where the functionalities required for potent bioactivity can readily incorporated. Recent studies report a similar pharmacophore as that described for linear peptides for the shorter cyclic dipeptides (65). Bottom row illustrate stereoisomers of cyclo(*N*-Bip-Arg-*N*-Bip-Arg) which display difference in bioactivity due to their ranging ability to form stable amphiphilic solution structures (93).

2,5 diketopiperazines (DKPs) are cyclic dipeptides and are regarded as privileged structures with the ability to bind to a range of natural receptors (90). 2,5-DKPs present a near ideal scaffold for the development of novel antimicrobial compounds, as they are chemically accessible, highly stable, and amenable to extensive synthetic derivatisation at up to six positions to develop versatile bioactive compounds (69, 91, 92). In addition to freedom in terms of amino acid stereochemistry and choice of sidechains, up to four additional substituents can be further incorporated *via* different alkylation strategies (90, 91). Taking advantage of the readily available scaffold Labrière *et al.* recently reported the preparation of a series of 2,5-DKPs adhering to the design principles of the aforementioned linear tripeptides developed by Strøm and co-workers (65). Focussing mainly on diastereomeric mixtures, contribution from both charge and bulk were probed and the results showed that the evaluated DKPs exhibited similar or superior antimicrobial activity in comparison to structurally related linear peptides incorporating unnatural amino acids (65). An enantiopure analog was prepared and shown to display improved activity against all tested bacteria compared to the diastereomeric mixture. Additional work and improvement of the synthetic methodology (69) allowed Grant *et al.* to generate an enantiopure library of 2,5- DKPs to further investigate the role of stereochemistry on the effect of these compounds (93). The stereoisomers of cyclo(*N*-Bip-Arg-*N*-Bip-Arg) were prepared and a clear dependence on the ability to form stable amphiphilic structure for a high activity was verified through spectroscopic and modelling experiments (**Figure 6**) (93).

## Ultrashort Lipopeptides

**Figure 7:**
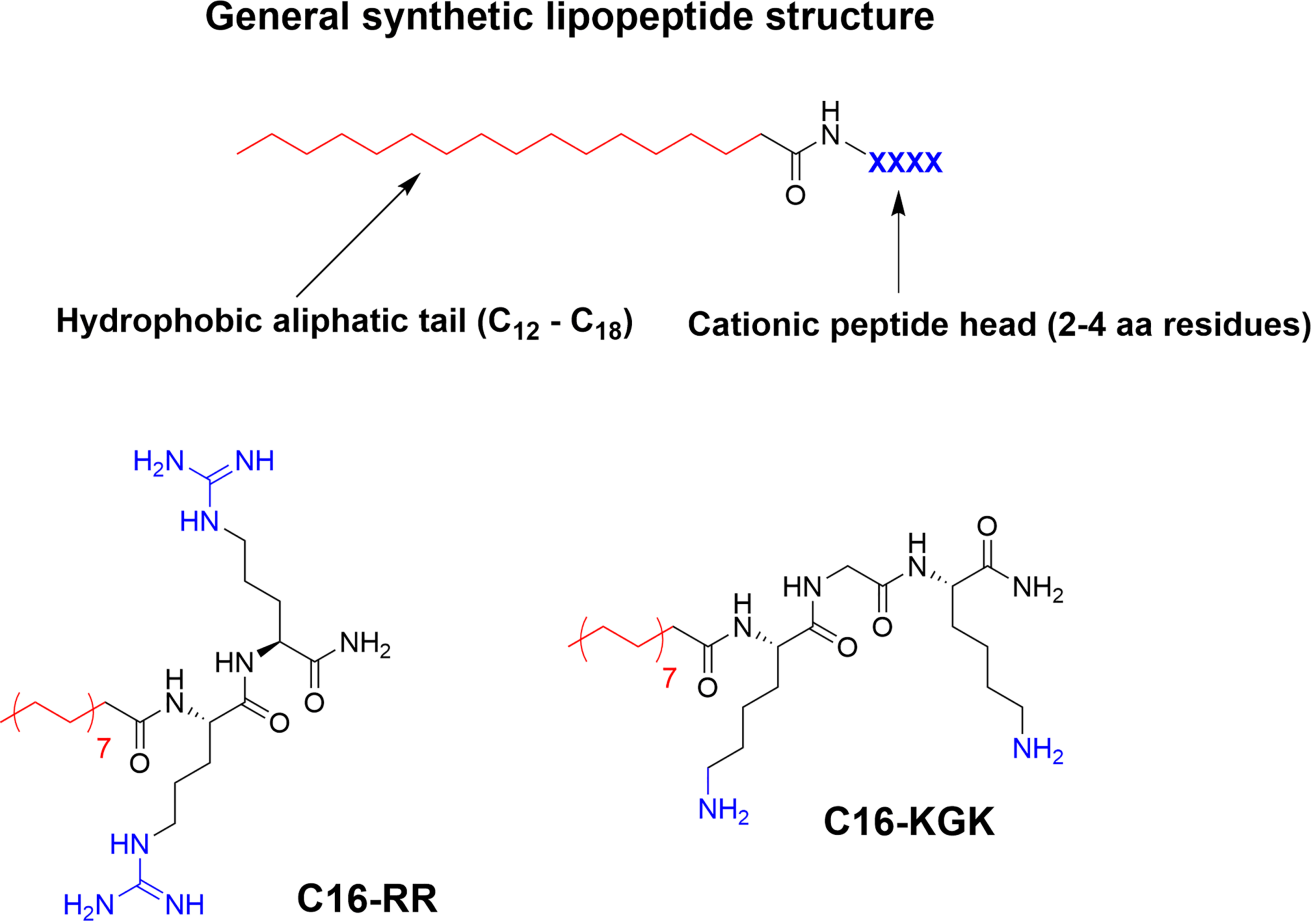
General structure of a USLiP and bioactive examples with a cationic di- to tripeptide providing the cationic charge to sufficiently balance the N-terminal fatty acid tail to generate potent antimicrobial amphiphiles (100, 102).

Incorporation of larger hydrophobic residues and unnatural sidechains has been shown to be highly beneficial for antimicrobial activity (6, 57). An alternative way to incorporate this hydrophobic element is though *N*-terminal modification with fatty acids to generate so called lipopeptides (LiPs) (70). Natural antimicrobial LiPs exist and are nonribosomally produced by some bacteria and fungi (70, 94). This class of antibiotics has demonstrated high activity against multidrug-resistant microorganisms with daptomycin and caspofungin serving as examples of approved antimicrobial LiPs in clinical use (95–97). Natural LiPs are generally smaller than AMPs with short peptide chains composed of either six or seven amino acids coupled to an N-terminal C_8_-C_18_ fatty acid chain (70). As such they do not adhere to the antimicrobial pharmacophore but simplified *de novo* designed ultrashort lipopeptides (USLiPs) have been prepared (71, 98, 99). Shai and co-workers pioneered this area (72) by developing a series of highly potent USLiPs composed of four L and D amino acids with the general KXXK motif (71). The potency and cellular selectivity could be controlled *via* the alkyl chain length and peptide sequence (71, 100). Since their initial development shorter sequences and both di- and trimeric versions have been prepared (101–103) and they performed successfully *in vivo* in a mouse model of *Candida albicans* infection (102) (**Figure 7**). Effective USLiPs can be prepared with two cationic residues and they act synergistically together with beta-lactams and vancomycin *in vitro (* 104). Beneficial potentiation effects have also been reported for dilipidic tetrabasic lipopeptides in a series of recent report by the Schweizer group (105, 106). These compounds can effectively potentiate the effect of Novobiocin and Rifampicin against Gram-negative bacteria and the effect can be modified by *N*-methylation of the cationic residues (107). Employing alternative scaffold backbones such as α-AApeptides and α/γ-AA hybrid peptides have also been shown to yield efficient lipidated analogs (108, 109). The history and development of natural and synthetic lipopetides have been reviewed (70, 110) and their industrial potential and application has been recently described (111, 112).

## Peptoids

**Figure 8:**
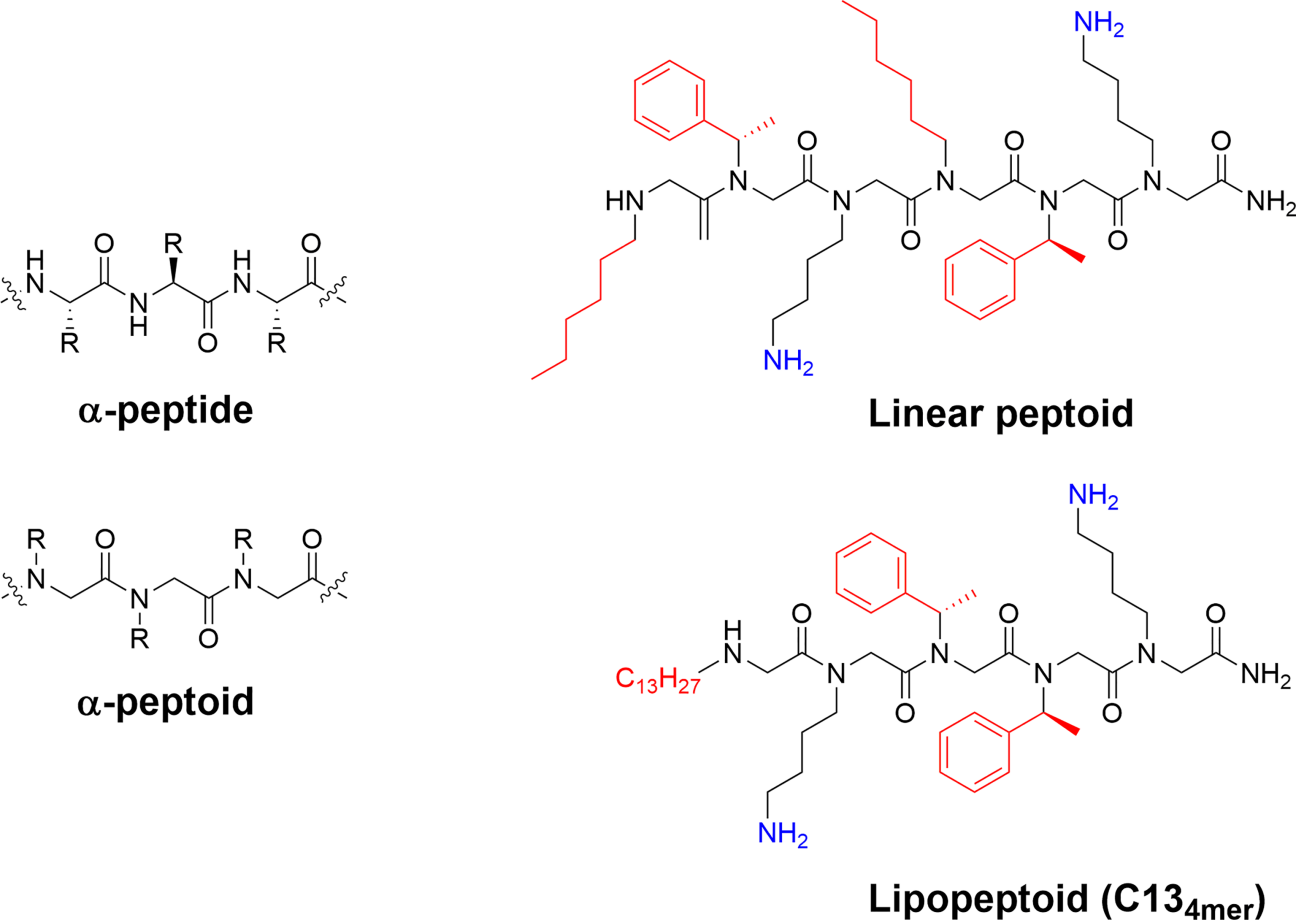
Structure and examples of antimicrobial peptoids. The difference between the backbone of α-peptides and α-peptoids allows for differences in structure and stability of the final compounds. The peptoid scaffold can be used to generate highly active antimicrobial compounds with the linear H-(*N*he-*N*spe-*N*Lys)_2_-NH_2_ and the lipopeptoid C13_4mer_ being active against *Mycobacterium tuberculosis* and ESKAPE pathogens (73, 116, 120).

The natural α-peptides can be efficiently mimicked by using oligo-*N*-substituted glycines with the sidechains attached to the backbone nitrogens instead of to the α-carbon (73). These peptoids are relatively easy to prepare synthetically and they offer an achiral backbone that is stable to proteolytic degradation. Despite a lack of amide hydrogens for internal hydrogen bonding formation, peptoids can be designed in an amphiphilic manner to adhere to the structural requirements reported for short linear antimicrobial peptides with the same chemical toolbox available to optimize their properties (113–115). Short peptoids have been shown to adopt various self-assembled structures (116, 117) and several potent larger antimicrobial helical peptoids and peptide/peptoid hybrids have been reported with efficacy towards drug-resistant bacteria (73, 114, 118, 119). In analogy to the α-peptides, peptoids can also be used as analogues to linear peptides to generate potent lipopeptoids by attachment of linear alkyl chains (116, 120–122) as shown in **Figure 8**. Furthermore, the incorporation of halogen atoms into peptoid structures have yielded active leads (115) against a range of ESKAPE pathogens (116). The development of anti-infective peptoids was recently reviewed in detail by Bicker and Cobb (73).

Longer α-AApeptides (123) and γ-AApeptides (124) have been used to prepare mimics of AMPs and it is expected that these scaffold can be used also for generating these shorter mimics in analogy to the peptoids (73).

## Bile Acid Derivatives (Ceragenins)

**Figure 9:**
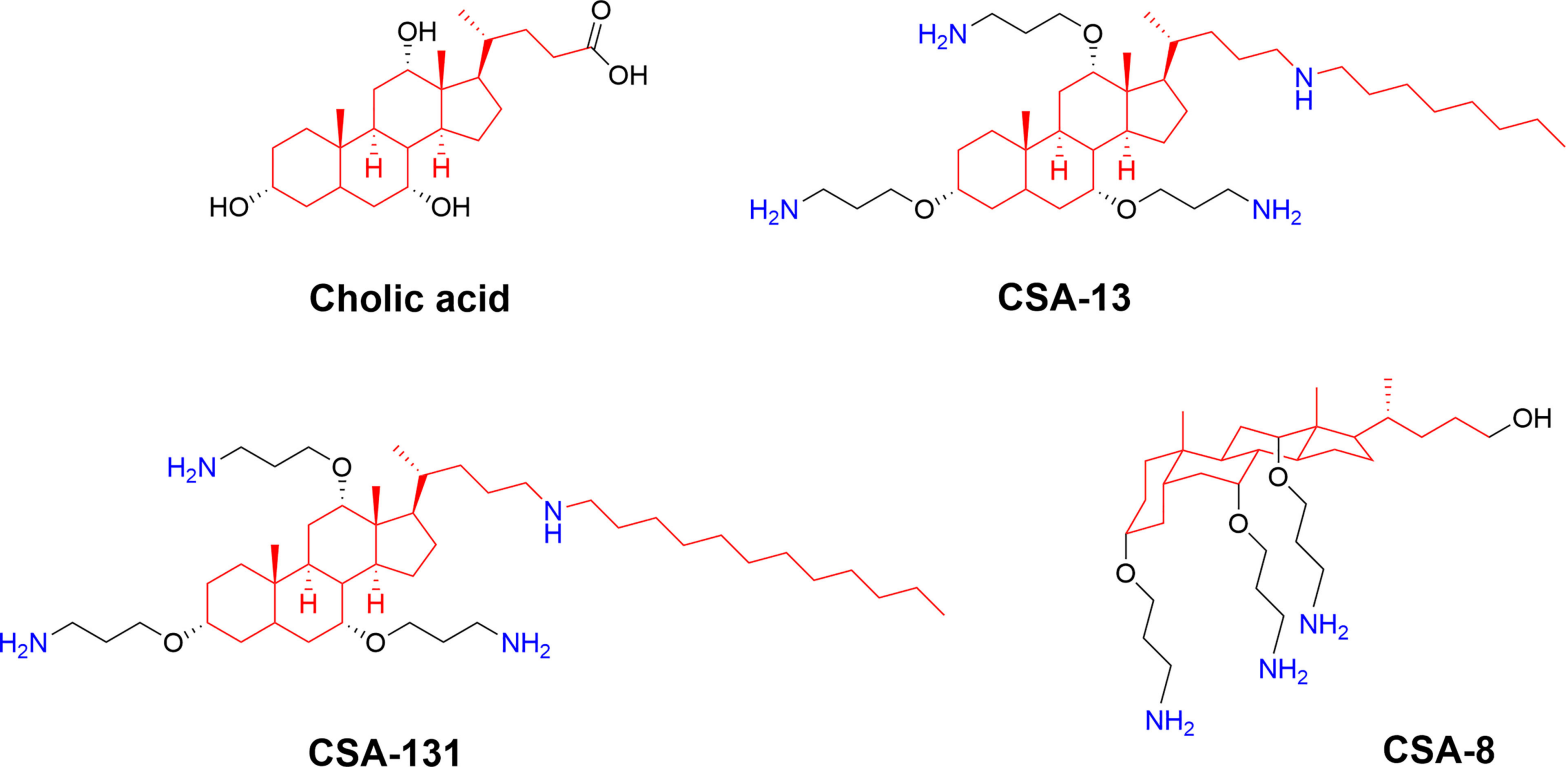
Structures of natural cholic acid and examples of the ceragenins CSA-13, and CSA-131 incorporating amine and alkyl modification. The perspective drawing of CSA-8 illustrates the facial amphiphilicity of the final compounds which is key for bioactivity (74).

The naturally amphiphilic cholic acid is produced endogenously to solubilize lipids as part of the digestion process and several studies have described ways to utilize the scaffold to generate compounds designed for membrane interactions (74, 75). By further fine-tuning the amphiphilic nature of the cholic acid scaffold, Savage and co-workers developed potent nonpeptidic mimics of cationic AMPs, initially known as cationic steroid antibiotics (76), but later renamed “ceragenins” (74). The ceragenins are designed to mimic the facial cationic morphology of natural AMPs and the three free hydroxyls and the carboxy group offer means to develop a range of different ceragenins with optimal activity and cellular selectivity by incorporation of both basic and bulky substituents (75) (**Figure 9**). The ceragenins CSA-13 and CSA-131 were equipped with an alkyl chain to increases their ability to interact with lipid A and they were particularly active against both bacteria and fungi (74, 125, 126). The CSA-13 ceragenin was initially developed towards clinical use by Ceragenix Pharmaceuticals and there is currently a significant focus on developing surface coatings and nanoparticles incorporating leaching and immobilized ceragenins (127) for prevention of biofilm formation using the second generation CSA-131 lead (128). The Cerashield™ Endotracheal Tube (the CeraShield ETT) is currently being developed by N8 Medical for complications experienced by mechanically-ventilated patients and it has been evaluated in human clinical trials (NCT03716713). This development is parallel with the development of AMC-109 for surface protection by Amicoat AS (62). A plethora of different substituents has been investigated on the bile acid scaffold, such as amino acids (129) and bile-based antimicrobials were recently reviewed by Lin et al. (75).

## Synthetic Mimics of Antimicrobial Peptides (SMAMPs/smHDPs)

**Figure 10:**
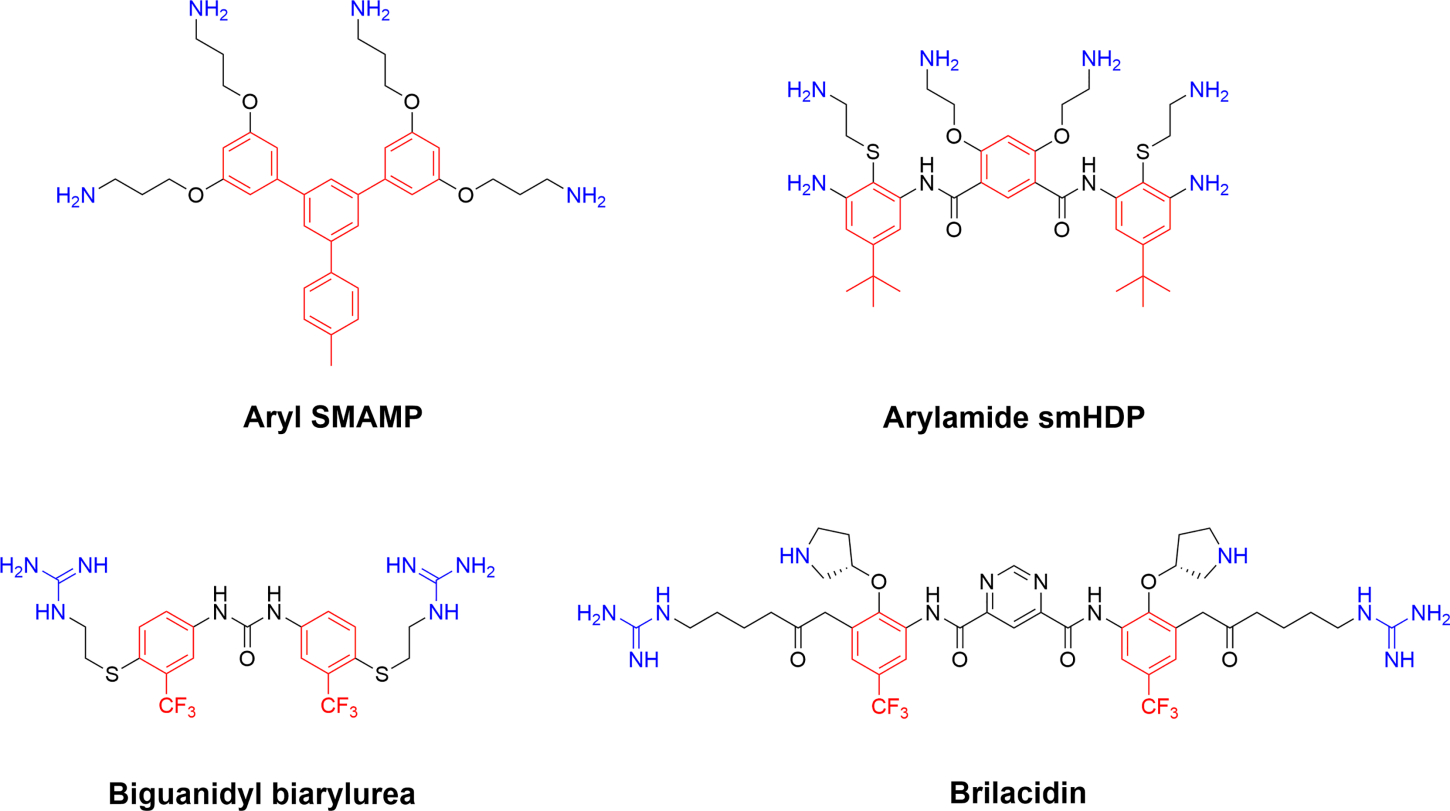
Representative early SMAMPs illustrating the chemical diversity available to generate cationic synthetic amphiphilic compounds adhering to the AMP pharmacophore (40, 133). Briliacidin is currently being developed by Innovation Pharmaceuticals for a range of conditions such as ulcerative proctitis, oral mucositis, ABSSSI and as inhibitors for SARS-CoV-2.

The realisation that it is possible to move away from the oligomeric “amino acid scaffold” of α, β-peptides and peptoids for the generation of potent antimicrobial compounds has spurred considerable research into abiotic scaffolds fulfilling the same structural requirements. The development of these diverse compounds was initially led by Tew and Scott and their work has yielded several different effective synthetic mimics of antimicrobial peptides (SMAMPs) and larger polymeric assemblies (40, 130). The diverse SMAMP scaffolds have been designed not to duplicate the natural peptide structures but in an attempt to generate smaller compounds with better pharmacokinetic and tissue distribution properties that can be prepared more cheaply on large scale (40). Whilst producing small compounds displaying both cationic and hydrophobic functionalities can be seen as chemically straightforward, the challenge lies in maintaining both cellular selectivity and therapeutic index. The tools at hand are often a choice between amines, guanidine groups and quaternary ammonium groups balanced with appropriate hydrophobic elements. Much of the research has been focussed on antibiotic-like substances but efforts to include them into different functional polymers have also been made by Lienkamp and co-workers (131, 132). Selected successful SMAMP scaffolds include aryl SMAMPs (133) meta-phenylene ethynylene (77), arylamides (134, 135), diphenyl dibenzopyrole (136), biguanidyl biarylurea (137), and porphyrin (138) (**Figure 10**). A particular promising arylamide SMAMP based on a central pyrimidine (PMX207) was shown to be promising leads against chloroquine resistant *Plasmodium falciparum (* 137) and further work on the same scaffold (139) produced PMX30063 which is now under clinical development as Brilacidin by Innovation Pharmaceuticals Inc. (formerly Cellceutix) (40). Brilacidin has demonstrated potent bactericidal activity against drug-resistant and drug-susceptible strains of multiple Gram-negative and Gram-positive pathogens with a membrane depolarization mode of action similar to daptomycin (140) and *via* immunomodulation (140, 141). Several initial clinical trials on brilacidin for the treatment of acute bacterial skin and skin structure infections (ABSSSI) have been conducted and the lead displayed low toxicity and was well tolerated (40). Briliacidin has also been recently reported to be highly active against SARS-CoV-2 in cell culture (141, 142) which illustrates the potential for expanded work on this class of AMP mimics, which has been reviewed by Scott & Tew in 2017 (40).

**Figure 11:**
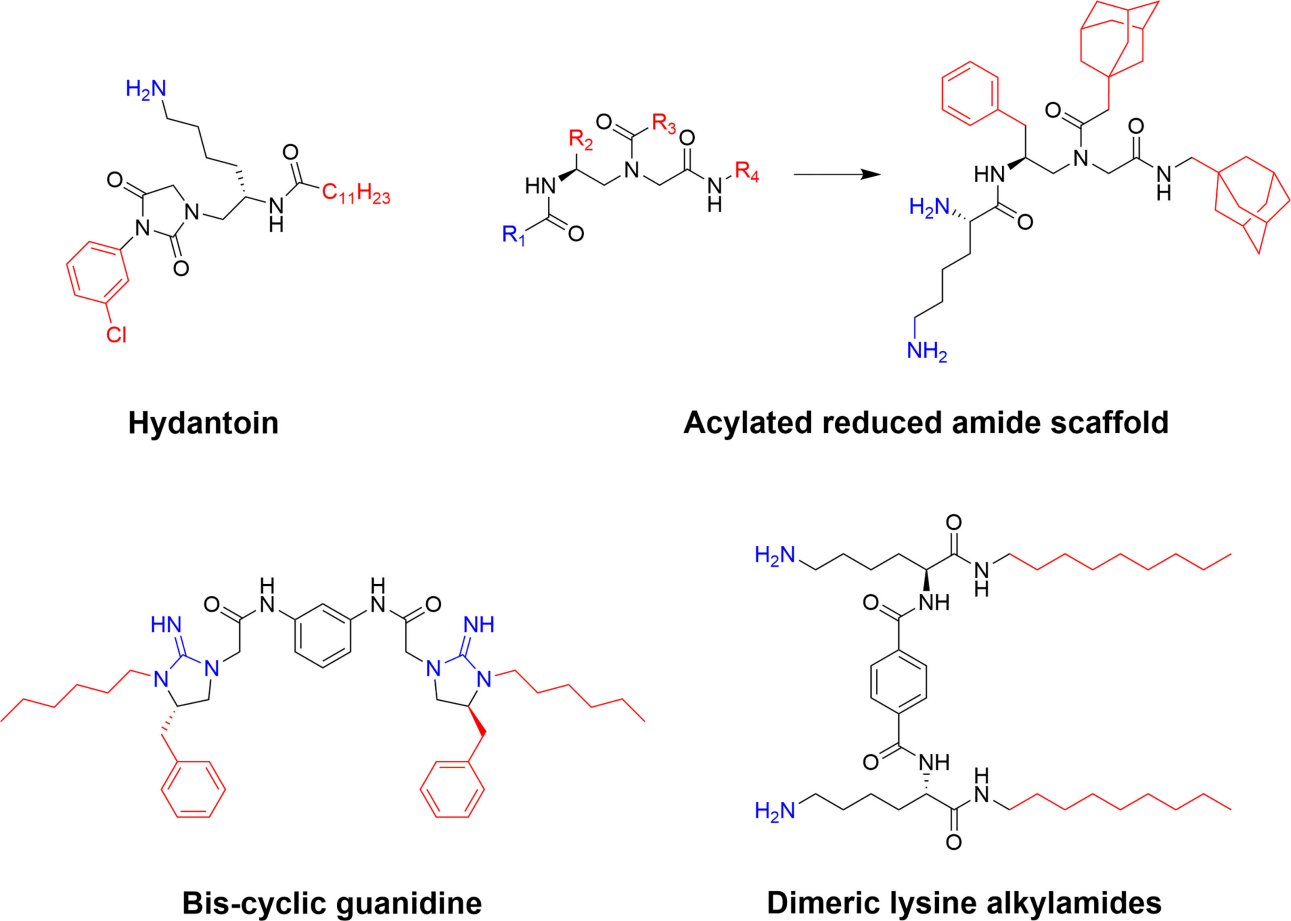
Examples of potent SAMP scaffolds and lead compounds developed by Cai and co-workers (78). Several of these compounds, such as the hydatoin and the cyclic guanidine derivative, display good antimicrobial activity both *in vitro* and *in vivo* (143, 144).

The group of Cai has also recently prepared an extensive series of different SMAMPs (or mimics of host-defence peptides, HDPs) with a focus on synthetic simplicity (78). A series of disubstituted hydantoins were prepared by combining the structure of the hydantoin based antibiotic nitrofurantoin with the structural element needed for bacterial membrane activity (143). The lead compound of the series displayed low micromolar MIC-values against resistant bacteria and exhibited potent *in vivo* efficacy for the treatment of lungs infected with MRSA in a rat model (143). Further work included small dimeric cyclic guanidine derivatives active against both multidrug-resistant Gram-negative and Gram-positive bacteria. The activity *in vitro* was subsequently verified *in vivo* using a MRSA-infected thigh burden mouse model (144). Additional rationally designed SAMP scaffolds evaluated include lysine *N*-alkylamides *(* 145) reduced amide-based compounds (146) and a range of others based on aromatic linkers (147–149) which have produced effective antimicrobial compounds (**Figure 11**).

**Figure 12:**
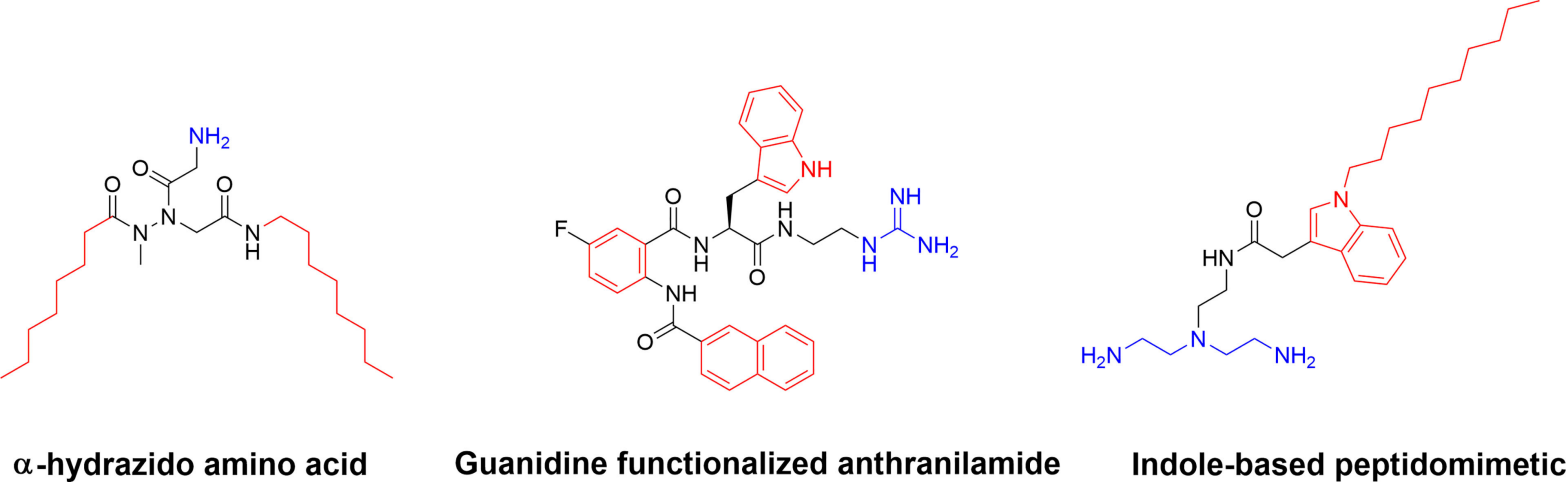
Examples of small and synthetically simple SAMPs illustrating that it is possible to also achieve good antibacterial activity with as single positive charge in some scaffolds.

Additional SMAMP-type compounds include the recently reported substituted α-hydrazido amino acids developed by Amabili and co-workers (150). The mono-charged α-hydrazido amino acids can be regarded as mimics of β-amino acids and they were designed with a range of both N- and C-terminal lipophilic groups to yield potent amphiphilic compounds (150). The versatile bicyclic norbornane scaffold has also been used for the generation of SMAMPs (151). Pfeffer and co-workers developed norbornane bisether diguanidines that were shown to display submicromolar inhibitory activity against both *MRSA* and vancomycin-intermediate *S. aureus* strains (152, 153). The cellular selectivity of the norbornane compounds could be controlled by choice of hydrophobic substituent (153). Building on their work on antibacterial biphenyl compounds (154), Kumar and co-workers also reported biofilm disrupting guanidine functionalized anthranilamides (155) with good selectivity for bacterial cells over mammalian MRC-5 cells *in vitro (* 155). Both Yang et al. (156) and Chen et al. (157) have developed synthetically simple indole based antimicrobials. By using different lipophilic *n*-acyl side chains at position 1 and a positively charged unusual azepanyl moiety at position 3 a large number of analogs were prepared and shown to display activity against *Mycobacterium bovis* and *M. tuberculosis* with both metabolic stability and cellular selectivity (156). Taking a similar approach and starting with ethyl 3-indoleacetate as a cheap starting material, Chen and co-workers recently designed and synthesized membrane-targeting indole-based antimicrobial peptidomimetics. The hydrophobic groups included isoprenyl, geranyl, heptenyl groups and the indole scaffold, while the cationic groups were composed of a range of amino acids or aliphatic amines and guanidines (157). Several active compounds were prepared, and a lead compound displayed high potency against Gram-positive bacteria in a murine model of bacterial keratitis. It was also shown to be more efficient than vancomycin. In addition, the lead compound was shown to not be affected by physiological concentrations of monovalent, divalent, or trivalent cations (**Figure 12**) (157).

**Figure 13:**
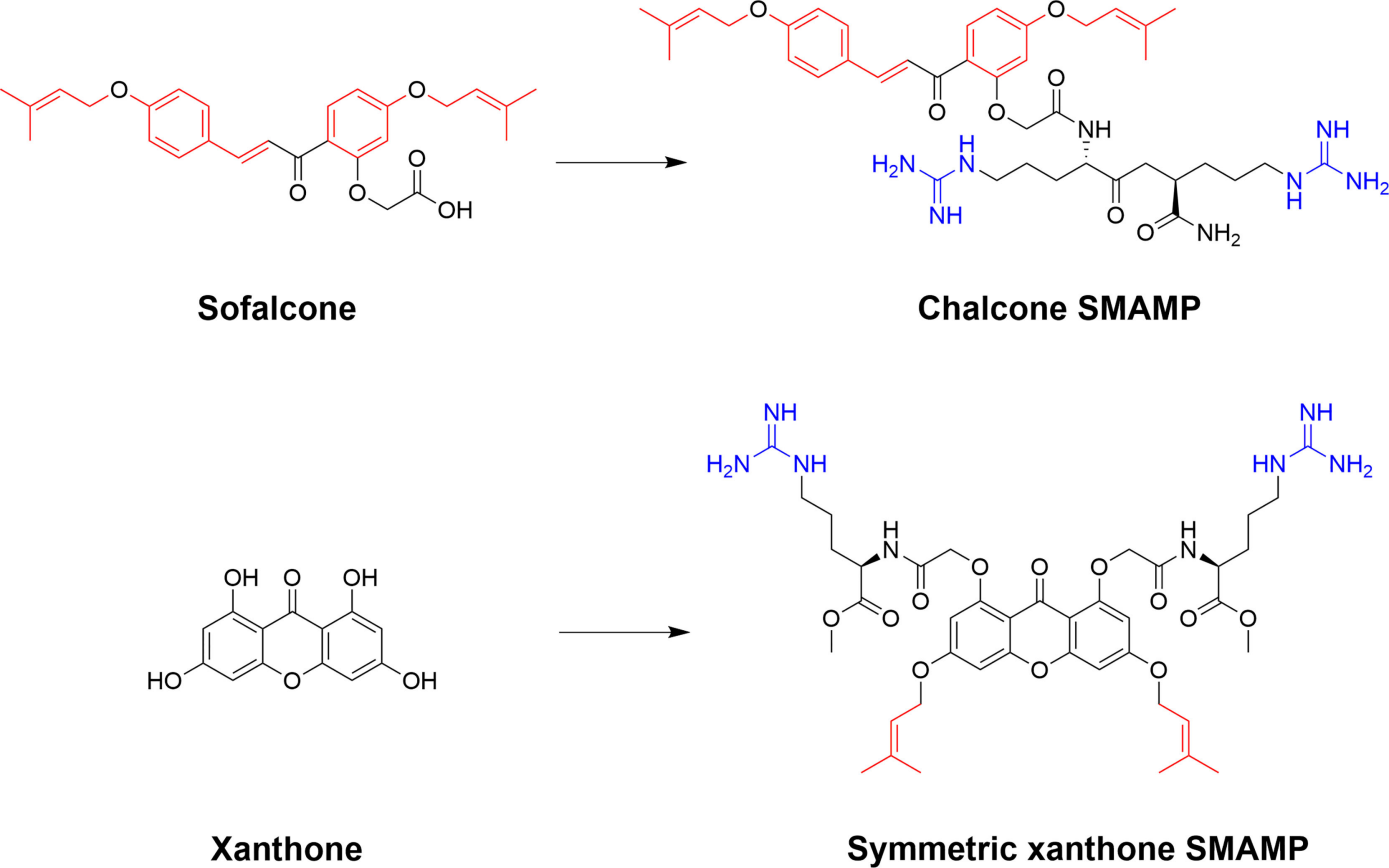
Examples of ways to functionalize natural scaffolds from plants into potent SMAPs. Both chalcones and xanthones offer ample opportunity for synthetic modification to introduce both cationic and hydrophobic residues to achieve the essential balance for high antimicrobial activity and low mammalian cytotoxicity (160, 161).

The phenolic chalcones is a diverse family of bioactive natural products with ranging bioactivities that has been used as scaffolds to yield improved synthetic analogs (158, 159). The scaffolds are readily available for semisynthetic modifications and Lin *et al.* recently used the isoprenyl chalcone derivative sofalcone as a scaffold to generate potent SMAMPs (160). Sofalcone, prepared from soforadine isolated from the root of the plant *Sophora subprostrata*, was functionalized with a range of cationic residues and the strongest activity was observed for sofalcone coupled to an RR-dipeptide (160). Previous related work on natural polyphenolic compounds from the group also include the generation of symmetrically substituted xanthone-based amphiphiles (79, 161). The substituted α-mangostin xanthone was used as a hydrophobic core to yield several potent symmetrical SMAMPs which were also effective *in vivo* in a mouse model of corneal infection by either *Staphylococcus aureus* or MRSA (79). Additional work by Lin et al. also include the design and semisynthesis of antimicrobial amphiphilic flavones (162) and coumarin derivatives (163). These examples illustrate how natural product scaffolds represent versatile cores for the design of efficient SMAMPs (**Figure 13**). *De novo* design of flavonoid-based AMP mimics was recently reviewed (164).

## Structure-Activity Relationship

The mimics included in this review were selected based on their adherence to the 2 + 2 pharmacophore and an optimized balance between the number of cationic charges and the hydrophobic elements. With careful design, the compounds can be tuned to display similar activity against both Gram-positive and Gram-negative bacteria (6, 85). Several compounds have likely been designed without specifically taking this pharmacophore into account but have nevertheless provided compounds adhering to the proposed design principle of Strøm and co-workers (6, 32).

The design of these mimics generally falls into one of three different approaches: 1. the oligomeric approach using amino acids or analogous monomers to assemble a small chain, 2. the scaffold approach in which a natural scaffold is substituted to yield highly active compounds (*e.g*. ceragenins) or 3. *de novo* design of purely synthetic compounds (SMAMPs). Our current report illustrates that each approach can be successfully employed to yield very effective antimicrobial compounds suited for clinical development and it is clear that the researcher is not limited to the native AMPs to generate new promising antibiotic leads.

The bioactivity of the majority of native AMPs hinges on a stable amphiphilic structure to allow for optimized membrane interaction (31, 82, 165). AMPs in general comprise up to 50 amino acids and these polypeptides are thus of sufficient size to allow folding into different bioactive motifs (19, 24, 31). The amphiphilicity of an AMP is a reflection of the relative abundance of hydrophilic and hydrophobic residues or domains within the AMP. It is therefore a good descriptor of the balance between the cationic and hydrophobic residues, not just within primary sequence level, but also in terms of the 3-dimensional structure of the AMPs (165). The exact mode of action is expected to depend on the sequence/structure and target species and are often not reported in detail beyond illustrating membrane insertion or disruption (93, 166). Selected mimics have been shown to be active also against marine microorganisms which further suggest multiple modes of action given the spread in cell surface and membrane composition (6, 69, 167).

While our review has illustrated that several synthetic and natural scaffold can be employed to generate improved minimized mimics of AMPs, the majority of the scaffolds are too small to adopt the secondary structures of the native counterparts. Despite the limitations in the ability to form intramolecular bonds and structures stable in solution, increasing evidence suggest that significant gains in activity can be obtained by careful molecular design permitting, or locking, the compounds in facial amphiphilic structures also on this scale (39). Such structures can be obtained by careful sequence optimization or by tuning the stereochemistry. This has been exemplified for numerous of the scaffolds previously described including the tripeptides (166), SMAMPs (133), DKPs and α-hydrazido acids (150). Most studies on these types of small AMP mimics claim that the lead compounds are “amphiphilic” but only a few provide any structural or quantifiable physicochemical data in support, which is also likely dependent, to some extent, on the challenges associated with obtaining crystals.

**Figure 14:**
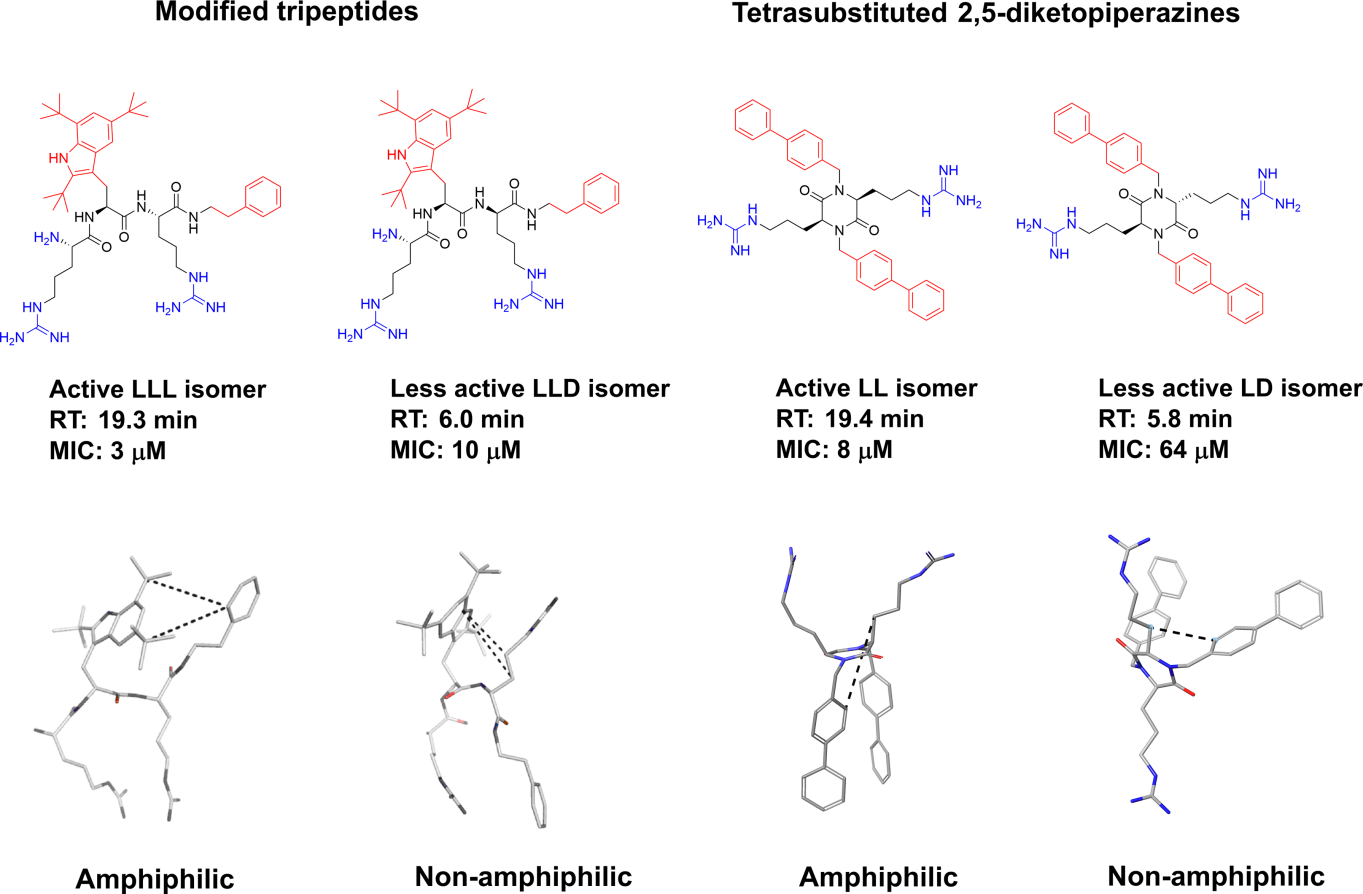
Graphical illustration of the role of stereochemistry on structure and antimicrobial activity of short, modified tripeptidic AMPs and tetrasubstituted 2,5-DKP. Making mixed analogs disrupted the amphiphilicity and creates more hydrophilic and less activity compounds (93, 166). Bottom left structures of LTX-109 are reprinted with permission from Isaksson et al (166). J Med Chem 2011 Vol. 54 Issue 16 Pages 5786-95. Copyright 2011 American Chemical Society.

Tew and co-workers studied the role of amphiphilicity for a series of SMAMPs by incorporating a polar amide bond between the hydrophobic residues. The integy moment (IW) was used to quantify the amphiphilicity of the SMAMPs and confirmed its necessity for the design of optimal SMAMPs (133). This disruption of the amphiphilicity was also reflected in the hydrophobicity of the SMAMPs (133). Only a few other selected published studies have probed the amphiphilicity experimentally. For oligomeric compounds, stereochemistry is a property that is readily tuneable to observe differences in 3D-structures. For this purpose, Isaksson and co-workers prepared all the stereoisomers of LTX-109 and studied the effect on antimicrobial effect (166). It was shown that all L- and all D- isomers retained a high antimicrobial activity while mixed isomers performed poorer in the microbial assays. To develop an understanding of the underlying mechanism the isomers were assessed using nuclear magnetic resonance (NMR) spectroscopy and molecular dynamics (MD) simulations in aqueous solution and in phospholipid bilayers. It was shown that the bioactive compounds were able to adopt a stable amphiphilic structure which was disrupted upon changes in the stereochemistry. This was not only apparent from the differences in antimicrobial activity but also in the solubility and retention time of the compounds. The bioactive amphiphilic compounds were significantly more hydrophobic, and this provided additional support for stable bioactive solution conformations (166). A similar study was recently reported by Grant and co-workers, and they observed similar phenomena for the tetrasubstituted 2,5-DKP scaffold (93). In their study the difference in elution time for the mixed isomers was also strongly reflected in the antimicrobial activity and NMR and MD experiments supported the formation of an optimized amphiphilic bioactive structure (**Figure 14**) (93).

A plethora of natural and unnatural cationic and hydrophobic building blocks have been employed to generate bioactive compounds and a move from the traditional amino acid-like scaffold provides the chemist with additional freedom to toggle these substituents further. It is clear that several compounds with good efficiency can be obtained slightly outside the 2 + 2 pharmacophore with examples such as the hydantoins and α-hydrazido amino acids that are active despite only displaying a single cationic residue (143, 150). In general, a higher number of charges appear to be needed *e.g*. a single lysine attached to palmitic acid (16 carbon atoms) does not show any activity (70). The nature of the cationic group is important (168), and many studies generally report increased activity for AMPs displaying guanidine groups over primary amine, histidine and ammonium groups (6, 34, 169, 170). The choice of cationic groups can also have effects of the toxicity of the compounds with several short compounds reporting higher toxicity for compounds incorporating amine substituents (79, 155, 171). It has been established that the exact number of hydrophobic residues is not as crucial and it is rather dependent on the total hydrophobic volume and its optimized molecular distribution (6, 65). Adding more hydrophobic residues usually increases the antimicrobial activity but also at a cost of lowered cellular selectivity (63, 64, 82). Adding too much hydrophobicity renders the compounds inactive, either due to aggregation or poor solubility and has been seen in several studies (69, 149, 172). The optimal balance between charge and hydrophobicity differs depending on scaffold studied and it has not been quantified much further than the 2 + 2 pharmacophore for the compounds covered in this review.

## Toxicity and Challenges

From a design perspective, an optimized combination between charge and hydrophobicity is needed. This allows the compounds to obtain the desired amphiphilicity in order to be antimicrobial and membrane active but also display sufficient selectivity and membrane distinction as several AMPs and their shorter mimics are toxic (82). Many of these described compounds have been designed to offer cheap and simple alternatives to AMPs to circumvent some of the limitations faced by natural peptide sequences for combating multidrug-resistant and pandrug-resistant bacterial isolates. Being mimics of cationic AMPs also potentially mean challenges associated with inactivation by high salt concentrations and divalent cations (173), even though studies describing a high salt tolerance and maintained function exist (157, 174). While impressive *in vitro* and *in vivo* activity profiles have been reported over the recent years, and several AMP mimics being in clinical development, their clinical safety is less understood. Numerous peptides are approved as drugs (1) and the peptide drug pipeline has been established for many areas. For AMPs, and these synthetic analogs in particular, there are several gaps in the clinical understanding particularly in the area of systemic toxicity (18).

## Conclusion

AMPs represent a broad class of diverse natural compounds with great promise for development of novel antibiotics with a lower likelihood of inducing microbial resistance. The clinical progression of AMP leads towards actual real-life use is nevertheless currently hampered (17) with only a small number of compounds on the market targeting a limited number of conditions, due to poor uptake and toxicity (17, 175). The AMPs in use are often small, constrained and contain modifications and noncanonical residues providing them with improved pharmacokinetics properties (30). This observation illustrates key natural methods to produce leads with a higher likelihood of clinical success by synthetic modifications, cyclisation and truncation (176, 177). In the current review we highlight how several of these means have been employed to address the challenges associated with clinical use of native AMPs by developing simplified synthetic mimics of AMPs. It becomes clear, when going through the literature, that ample room for making active and selective low molecular weight AMP mimics room exists, as illustrated by the many highly active mimics reported here. A careful design is nevertheless needed to generate compounds with a sufficient safety profile. These compounds can be cheaply produced in large amounts and with three diverse leads in clinical trials it is expected that these types of small synthetic mimics will play an important role in the future management of microbial infections. Recent studies on synergistic effects in combination with traditional antibiotics, further highlight the imminent role these compounds may have (103, 106, 178, 179). Collectively, it is shown that a high antimicrobial activity can be obtained using diverse low molecular weight compounds and it is clear that shape matters more than size for optimal activity.

## Author Contributions

Conceptualization: JS, NM, and CS; Investigation: JS, NM, and CS; Preparation of original draft: JS; Review and editing: JS, NM, and CS; Visualization: CS; Project administration: JS; Funding: JS and CS. All authors have read and agreed to the submitted version of the manuscript.

## Conflict of Interest

The authors declare that the research was conducted in the absence of any commercial or financial relationships that could be construed as a potential conflict of interest.

## Publisher’s Note

All claims expressed in this article are solely those of the authors and do not necessarily represent those of their affiliated organizations, or those of the publisher, the editors and the reviewers. Any product that may be evaluated in this article, or claim that may be made by its manufacturer, is not guaranteed or endorsed by the publisher.
